# Attention‐deficit/hyperactivity disorder polygenic risk score and incidence of cognitive impairment in older adults: A prospective multicohort study

**DOI:** 10.1002/alz.71770

**Published:** 2026-10-15

**Authors:** Douglas Teixeira Leffa, Guilherme Bauer‐Negrini, Livia Amaral, Bruna Bellaver, Pamela C. L. Ferreira, Guilherme Povala, Firoza Z. Lussier, João Pedro Ferrari‐Souza, Ann D. Cohen, Dana L. Tudorascu, Samuele Cortese, Luis Augusto Rohde, Brooke S. G. Molina, Derek Archer, Tharick A. Pascoal

**Affiliations:** ^1^ Department of Psychiatry University of Pittsburgh Pittsburgh Pennsylvania USA; ^2^ Neuroscience Unit, Hospital Moinhos de Vento Porto Alegre Rio Grande do Sul Brazil; ^3^ Department of Biostatistics University of Pittsburgh Pittsburgh Pennsylvania USA; ^4^ Developmental EPI (Evidence synthesis, Prediction, Implementation) Lab, Centre for Innovation in Mental Health, School of Psychology, Faculty of Environmental and Life Sciences University of Southampton Southampton UK; ^5^ Clinical and Experimental Sciences (CNS and Psychiatry) Faculty of Medicine University of Southampton Southampton UK; ^6^ Hampshire and Isle of Wight Healthcare NHS Foundation Trust Southampton UK; ^7^ New York University Child Study Center, Hassenfeld Children's Hospital at NYU Langone New York New York USA; ^8^ DiMePRe‐J‐Department of Precision and Regenerative Medicine‐Ionian Area University of Bari “Aldo Moro” Bari Italy; ^9^ ADHD Outpatient Program & Development Psychiatry Program Hospital de Clínicas de Porto Alegre Porto Alegre Brazil; ^10^ National Institute of Developmental Psychiatry & National Center for Innovation and Research in Mental Health São Paulo Brazil; ^11^ Medical Council UNIFAJ & UNIMAX Indaiatuba Brazil; ^12^ Departments of Psychiatry, Psychology, Pediatrics, Clinical and Translational Science University of Pittsburgh Pittsburgh Pennsylvania USA; ^13^ Vanderbilt Memory and Alzheimer's Center Vanderbilt University School of Medicine Nashville Tennessee USA; ^14^ Department of Neurology Vanderbilt Health Nashville Tennessee USA; ^15^ Vanderbilt Genetics Institute Vanderbilt Health Nashville Tennessee USA; ^16^ Vanderbilt Brain Institute Vanderbilt Health Nashville Tennessee USA; ^17^ Department of Neurology University of Pittsburgh Pittsburgh Pennsylvania USA

**Keywords:** alzheimer's disease, alzheimer's disease sequencing project, amyloid beta, attention‐deficit/hyperactivity disorder, cognitive aging, cognitive impairment, dementia, mild cognitive impairment, older adults, polygenic risk score

## Abstract

**INTRODUCTION:**

Preliminary evidence suggests a link between attention‐deficit/hyperactivity disorder (ADHD) and later‐life cognitive impairment. Whether genetic risk for ADHD, estimated using an ADHD polygenic risk score (ADHD‐PRS), is associated with incident cognitive impairment (mild cognitive impairment or Alzheimer's disease dementia) remains unknown.

**METHODS:**

We analyzed 7761 cognitively unimpaired (CU) older adults with whole‐genome sequencing from eight cohorts participating in the Alzheimer's Disease Sequencing Project (ADSP).

**RESULTS:**

During follow‐up, 1347 participants developed cognitive impairment. Ten‐year cumulative incidence was 33.8% in the highest ADHD‐PRS tertile versus 23.4% in the lowest. In Cox models, higher ADHD‐PRS remained associated with greater risk of cognitive impairment after adjustment for education, apolipoprotein E (*APOE)* ε4 status, or vascular risk. PRSs for other major psychiatric disorders were not associated with cognitive impairment in the same analytic framework. In a subsample with biomarker data, the association was not modified by baseline amyloid‐β status.

**DISCUSSION:**

These findings support ADHD‐PRS as a novel correlate of cognitive impairment and vulnerability in aging.

## Highlights

- Genetic risk for ADHD was estimated using PRSs.
- Across eight cohorts comprising 7761 participants, higher ADHD‐PRS was associated with greater risk of incident cognitive impairment.
- This association remained after additional adjustment for education, *APOE* ε4 status, or vascular risk.
- PRSs for other major psychiatric disorders were not associated with cognitive impairment in the same analytic framework.
- Baseline Aβ status did not modify the association between ADHD‐PRS and cognitive impairment.

## 1 BACKGROUND

Dementia affects more than 55 million people worldwide, yet the full landscape of factors contributing to late‐life cognitive decline remains incompletely understood.^1^ Attention‐deficit/hyperactivity disorder (ADHD) is a neurodevelopmental disorder that affects approximately 5% of school‐aged children and can persist throughout the lifespan, with prevalence estimates of ∼2.5% in young adults and ∼1% among adults older than 60 years.^2^ Emerging evidence suggests that ADHD is associated with a higher risk of mild cognitive impairment (MCI) and dementia.^3^, ^4^, ^5^, ^6^, ^7^, ^8^, ^9^, ^10^ Alzheimer's disease (AD) is the most common cause of dementia,^1^ and a large population‐based study reported elevated risk for AD among relatives of individuals with ADHD, suggesting a potential shared genetic risk.^11^

However, studies examining ADHD and later‐life cognitive impairment remain susceptible to residual confounding, including socioeconomic factors, health behaviors, and comorbid psychiatric and medical conditions.^12^ Furthermore, most studies rely solely on electronic health records to ascertain dementia and ADHD diagnosis,^3^, ^4^, ^5^, ^6^, ^7^, ^8^, ^10^ which may introduce bias due to differences in healthcare access, clinician recognition, and documentation practices. Crucially, existing cohorts of individuals diagnosed with ADHD in childhood have not yet reached an age where the risk of MCI or dementia can be adequately assessed,^13^ limiting the feasibility of rigorous longitudinal evaluation. These limitations have prevented firm conclusions from being drawn about whether ADHD represents a genuine risk factor for cognitive decline.

To complement this literature, genetic analyses have been proposed as an alternative approach. ADHD is highly heritable,^14^ with common genetic variants contributing significantly to risk.^15^ The ADHD polygenic risk score (ADHD‐PRS), which aggregates the effects of common variants identified in genome‐wide association studies (GWASs), has been associated with ADHD diagnosis and related traits.^16^ Therefore, ADHD‐PRS may provide a useful tool to investigate the relationship between ADHD genetic risk and late‐life cognitive outcomes. Recent studies have begun to explore whether higher ADHD‐PRS is associated with increased risk for cognitive decline in older age.^17^, ^18^, ^19^, ^20^ However, most are focused on cognitive test performance,^18^, ^19^, ^20^ and limitations in sample size and follow‐up duration restricted their ability to examine progression to clinical MCI or dementia.

To address these gaps, we aimed to evaluate whether genetic risk for ADHD, estimated with an ADHD‐PRS, is associated with the risk of progression from cognitively unimpaired (CU) status to MCI or AD dementia. We further performed sensitivity analyses to explore the role of potential confounders previously associated with both ADHD and dementia, including vascular risk factors,^1^, ^2^ assessed possible ADHD specificity by examining PRS for other psychiatric disorders, tested whether baseline amyloid beta (Aβ) status modifies this association, and evaluated the longitudinal relationship between ADHD‐PRS and cognitive performance.

**RESEARCH IN CONTEXT**

1. **Systematic review**: The authors reviewed the literature using traditional sources, including PubMed. A growing number of studies have examined ADHD in relation to later‐life cognitive impairment. Relevant works are appropriately cited.
2. **Interpretation**: Our results indicate that ADHD‐PRS, an estimate of genetic risk for ADHD, is associated with vulnerability to later‐life cognitive impairment. The absence of association with other psychiatric PRSs in the same models suggests a degree of specificity to ADHD. Clinically, the results suggest that individuals with higher ADHD genetic risk and those with diagnosed ADHD represent subgroups that may benefit from earlier/more frequent cognitive monitoring and targeted risk‐factor mitigation.
3. **Future directions**: Future studies should clarify mechanisms linking ADHD‐PRS to cognitive impairment through comprehensive biomarker phenotyping, follow childhood‐diagnosed ADHD cohorts into later life with repeated assessments, and determine whether individuals with clinically diagnosed ADHD or elevated ADHD‐PRS could benefit from earlier cognitive monitoring.

## 2 METHODS

### 2.1 Participants

We analyzed data from the Alzheimer's Disease Sequencing Project (ADSP), a large, multicohort collaborative initiative that integrates extensive longitudinal clinical characterization with large‐scale sequencing to accelerate discovery of genetic and other determinants of risk and resilience for AD and related dementias.^21^ We included individuals who were CU at baseline and had available age, sex, education, whole‐genome sequencing (WGS), *APOE* genotype, clinical assessments (including diagnoses and cognitive function), and vascular risk measures. Baseline was defined as the first visit at age ≥50 years with available clinical assessment. Included cohorts were Alzheimer's Disease Neuroimaging Initiative (ADNI), Estudio Familiar de Influencia Genética en Alzheimer (EFIGA), Rush Religious Orders Study (ROS) and Rush Memory and Aging Project (MAP), National Alzheimer's Coordinating Center (NACC), Texas Alzheimer's Research and Care Consortium (TARCC), Washington Heights/Inwood Columbia Aging Project (WHICAP), and Wisconsin Registry for Alzheimer's Prevention (WRAP). Further information on each cohort can be found in the Supplementary Material (Tables S1 and S2). Across all cohorts, written informed consent was provided by participants, and research was conducted in accordance with approved Institutional Review Board (IRB) protocols. Secondary analysis of these data was approved by the University of Pittsburgh IRB. Complete data were available for 7761 participants.

### 2.2 Polygenic risk scores

WGS data were obtained from ADSP Release 4,^22^ generated and processed by the Genome Center for Alzheimer's Disease (GCAD). Sequencing was performed at 10 centers using Illumina technology (more details can be found elsewhere^22^). Genetic data underwent the same robust quality control and imputation pipelines,^22^, ^23^ and fully quality‐controlled data were downloaded from the National Institute on Aging Genetics of Alzheimer's Disease Data Storage Site (NIAGADS). PRSs were computed using PRSice‐2 (version 2.3.5)^24^ based on GWAS summary statistics for ADHD^15^ and (in sensitivity analyses) autism spectrum disorder (ASD),^25^ bipolar disorder (BD),^26^ major depressive disorder (MDD),^27^ and schizophrenia (SCZ).^28^ After clumping to retain approximately independent variants (250‐kb window; LD *r* ^2^ = 0.1), we computed PRSs across 11 GWAS *p* value thresholds (1, 0.5, 0.4, 0.3, 0.2, 0.1, 0.05, 0.005, 0.0005, 5 × 10^−^^6^, 5 × 10^−^^8^). To reduce multiple testing and summarize liability robustly, we derived the first principal component (PC) across thresholds for each disorder‐specific PRS and used this score as the primary genetic liability measure.^29^ PRS values were standardized (z‐scores). To investigate populational structure, principal component analysis was conducted using PLINK 2.0.^30^

### 2.3 Clinical and cognitive outcomes

Clinical diagnoses and cognitive outcomes were obtained from the ADSP Phenotype Harmonization Consortium (ADSP‐PHC) Cognitive Dataset (NIAGADS accession number: NG00067.v18). The ADSP‐PHC was established to harmonize phenotype data across multiple cohorts. ADSP‐PHC diagnostic codes (CU, MCI, AD dementia) were downloaded from NIAGADS. All cohorts included in this study had their own protocols for clinical diagnosis determination, described in cohort publications (see Tables S1 and S2). These protocols typically involved multidisciplinary diagnostic review and applied standard criteria. For neuropsychological outcomes, ADSP‐PHC composite scores for memory, executive function, and language were downloaded from NIAGADS. The ADSP‐PHC developed robust methods to harmonize data from the different neuropsychological batteries across cohorts, with full details found elsewhere.^31^

### 2.4 Aβ biomarkers

A subset of participants had Aβ status from positron emission tomography (PET) and/or fluid biomarkers. Aβ PET data were obtained from ADSP‐PHC PET Dataset (NIAGADS accession number: NG00067.v18), processed by NIAGADS using magnetic resonance imaging‐free pipelines with tracer‐specific transformation of global Aβ standardized uptake value ratio to Centiloids. Further information on processing can be found in ADSP‐PHC‐FluidBiomarker_White‐Paper_2024.12.16.pdf, available via NIAGADS. PET‐derived Aβ positivity was defined using a single Centiloid threshold of 25 across tracers, using the harmonized variable (“PHC_AMYLOID_STATUS”) after quality control. Fluid‐derived Aβ status was obtained from the ADSP‐PHC Biomarker Dataset (NIAGADS accession number: NG00067.v18), which harmonized cerebrospinal fluid (CSF) Aβ42 data across cohorts. Further methodological information on the harmonization steps can be found in ADSP‐PHC‐FluidBiomarker_White‐Paper_2024.12.16.pdf, available via NIAGADS. In summary, fluid‐derived Aβ positivity was defined using Gaussian mixture models to determine cutoffs and represented by the harmonized variable “AT_class.” For analyses with individuals with Aβ data, we defined “Aβ‐baseline” as the first clinical assessment at age ≥50 years with an available Aβ measurement (PET and/or fluid). If both PET and fluid were available at the same qualifying visit, PET was prioritized. Time‐to‐event models in the biomarker subsample used this Aβ‐baseline as time zero.

### 2.5 *APOE* ε4 status

Apolipoprotein E (*APOE*) ε2/ε3/ε4 haplotypes were determined using rs429358 and rs7412. *APOE* ε4 status was modeled as ε4 carrier (≥1 ε4) versus non‐carrier.

### 2.6 Vascular risk score

Harmonized vascular risk scores were obtained from the ADSP‐PHC Cardiovascular Risk Factor Dataset (NIAGADS accession number: NG00067.v18). They were computed as the first PC summarizing cardiovascular risk indicators (binary history of hypertension, diabetes, heart disease, and stroke, as well as continuous body mass index) and represented by the harmonized variable “PHC_PC1_All.” Further methodological information can be found in ADSP‐PHC‐CardiovascularRisk_README_2024.12.16.docx, available via NIAGADS.

### 2.7 Statistical analyses

Kaplan–Meier methods were used to estimate the 10‐year cumulative incidence of cognitive impairment under right‐censoring and to visualize unadjusted differences across PRS tertiles (high vs low). We modeled progression from CU status to incident cognitive impairment (MCI or AD dementia) using Cox proportional hazards models, with ADHD‐PRS as a continuous predictor. Time was measured from baseline to first incident diagnosis, censored at last follow‐up or 10 years. This analytic window was selected to limit the influence of less stable estimates from sparse later follow‐up. Primary models adjusted for baseline age, sex, ancestry (first three PCs), and cohort. We then fitted three additional sensitivity models, each adding one of the following variables: education, vascular risk, or *APOE* ε4 status. To evaluate specificity, we repeated these models using PRS for other psychiatric disorders (ASD, BD, MDD, and SCZ) as predictors, applying the same modeling framework. In the biomarker subsample, we fitted analogous Cox models anchored at Aβ‐baseline and tested effect modification by Aβ status via an ADHD‐PRS × Aβ interaction term, additionally reporting Aβ‐stratified estimates. For continuous neuropsychological outcomes, we fitted linear mixed‐effects models to harmonized domain composites with fixed effects for ADHD‐PRS, time from baseline, their interaction (PRS × time), and covariates (baseline age, sex, ancestry PCs, and cohort), with participant‐specific random intercepts and random slopes for time. The PRS main effect was interpreted as baseline differences and the PRS × time interaction as differential rate of change. To evaluate whether the primary findings were influenced by diagnostic reversion after incident MCI/AD dementia, we identified participants who developed MCI or AD dementia and subsequently returned to CU status during the 10‐year analytic follow‐up window. We classified these participants as sustained CU reverters or transient CU reverters. Sustained CU reverters were defined as participants who returned to CU after incident MCI/AD dementia and remained CU at all subsequent visits. Transient CU reverters were defined as participants who returned to CU status after incident MCI/AD dementia but later reconverted to MCI or AD dementia. We then performed a sensitivity analysis in which sustained CU reverters were treated as nonevents and censored at their last available visit, whereas transient CU reverters were retained as events. All analyses were conducted in R (version 4.4.1) with two‐sided α = 0.05.

## 3 RESULTS

We analyzed data from 7761 CU participants from eight cohorts (Table 1). Mean (SD) baseline age was 70.97 (9.72) years and 5252 (67.7%) were women. Across follow‐up, we evaluated a total of 38,087 diagnostic assessments. Participants contributed a mean (SD) of 4.91 (2.88) diagnostic visits, and the median (interquartile range) observation time to incident cognitive impairment or censoring was 5.08 (1.99 to 9.47) years.

**TABLE 1:** Baseline demographic and clinical characteristics of the population.

Characteristic	Total sample (*N* = 7761)	ADNI (*N* = 481)	EFIGA (*N* = 790)	MAP (*N* = 190)	NACC (*N* = 4129)	ROS (*N* = 219)	TARCC (*N* = 625)	WHICAP (*N* = 357)	WRAP (*N* = 970)
Age at baseline, mean (SD), years	70.97 (9.72)	74.27 (5.77)	69.76 (8.18)	81.79 (5.18)	74.39 (7.56)	76.18 (6.33)	63.65 (7.79)	74.75 (5.24)	55.79 (4.76)
Sex, no. (%)									
Female	5252 (67.7)	250 (52.0)	516 (65.3)	133 (70.0)	2820 (68.3)	142 (64.8)	452 (72.3)	266 (74.5)	673 (69.4)
Male	2509 (32.3)	231 (48.0)	274 (34.7)	57 (30.0)	1309 (31.7)	77 (35.2)	173 (27.7)	91 (25.5)	297 (30.6)
Race, no. (%)									
American Indian or Alaska Native	49 (0.6)	1 (0.2)	0 (0.0)	0 (0.0)	40 (1.0)	0 (0.0)	3 (0.5)	5 (1.4)	0 (0.0)
Asian	55 (0.7)	8 (1.7)	1 (0.1)	0 (0.0)	12 (0.3)	0 (0.0)	32 (5.1)	0 (0.0)	2 (0.2)
Black or African American	1282 (16.5)	30 (6.2)	45 (5.7)	0 (0.0)	1068 (25.9)	0 (0.0)	0 (0.0)	95 (26.6)	44 (4.5)
Native Hawaiian or Other Pacific Islander	1 (0.0)	0 (0.0)	0 (0.0)	0 (0.0)	1 (0.0)	0 (0.0)	0 (0.0)	0 (0.0)	0 (0.0)
White	5467 (70.4)	436 (90.6)	116 (14.7)	190 (100.0)	2940 (71.2)	219 (100.0)	590 (94.4)	53 (14.8)	923 (95.2)
Other/unknown/more than one race	907 (11.7)	6 (1.2)	628 (79.5)	0 (0.0)	68 (1.6)	0 (0.0)	0 (0.0)	204 (57.2)	1 (0.1)
Ethnicity, no. (%)									
Hispanic or Latino	2175 (28.0)	15 (3.1)	775 (98.1)	0 (0.0)	463 (11.2)	1 (0.5)	625 (100.0)	288 (80.7)	8 (0.8)
Not Hispanic or Latino	5560 (71.6)	462 (96.0)	1 (0.1)	190 (100.0)	3663 (88.7)	218 (99.5)	0 (0.0)	65 (18.2)	961 (99.1)
Not available	26 (0.3)	4 (0.8)	14 (1.8)	0 (0.0)	3 (0.1)	0 (0.0)	0 (0.0)	4 (1.1)	1 (0.1)
Education, mean (SD), years	14.18 (4.76)	16.45 (2.65)	8.25 (5.77)	14.79 (2.88)	15.48 (3.20)	18.53 (3.46)	10.28 (4.71)	7.41 (4.43)	16.27 (2.78)
*APOE* ε4, no. carriers (%)	2382 (30.7)	139 (28.9)	213 (27.0)	33 (17.4)	1346 (32.6)	52 (23.7)	123 (19.7)	90 (25.2)	386 (39.8)
Vascular risk factors									
Vascular risk score, mean (SD)	0.08 (1.19)	−0.93 (0.73)	−0.18 (1.03)	0.39 (1.18)	0.18 (1.07)	0.63 (1.25)	0.37 (1.33)	0.98 (1.31)	−0.36 (1.29)
Hypertension, no. (%)	6358 (81.9)	243 (50.5)	663 (83.9)	184 (96.8)	3836 (92.9)	211 (96.3)	489 (78.2)	348 (97.5)	384 (39.6)
Diabetes, no. (%)	1699 (21.9)	18 (3.7)	205 (25.9)	40 (21.1)	856 (20.7)	52 (23.7)	240 (38.4)	154 (43.1)	134 (13.8)
Heart disease, no. (%)	2179 (28.1)	2 (0.4)	82 (10.4)	69 (36.3)	1335 (32.3)	105 (47.9)	110 (17.6)	172 (48.2)	304 (31.3)
Stroke, no. (%)	577 (7.4)	8 (1.7)	30 (3.8)	31 (16.3)	254 (6.2)	47 (21.5)	65 (10.4)	82 (23.0)	60 (6.2)
BMI (SD)	27.65 (5.65)	27.16 (4.79)	26.42 (4.97)	26.45 (5.45)	27.19 (5.40)	26.39 (5.17)	31.04 (6.29)	27.54 (4.89)	29.23 (6.41)
Composite score at baseline, mean (SD)									
Memory composite	0.66 (0.53)	0.83 (0.46)	0.34 (0.56)	0.35 (0.38)	0.67 (0.49)	0.46 (0.37)	0.52 (0.50)	0.28 (0.51)	1.08 (0.44)
Executive function composite	0.44 (0.81)	0.75 (0.48)	−0.58 (0.88)	0.47 (0.48)	0.57 (0.64)	0.54 (0.55)	−0.14 (0.62)	−0.53 (0.68)	1.10 (0.60)
Language composite	0.45 (0.71)	0.78 (0.53)	−0.40 (0.64)	0.34 (0.45)	0.69 (0.57)	0.39 (0.53)	−0.13 (0.55)	−0.37 (0.54)	0.67 (0.55)

*Note*: Values are mean (SD) for continuous variables and no. (%) for categorical variables. Percentages are calculated within each cohort. Age and education are in years; BMI is in kg/m^2^. *APOE* ε4 carriers are participants with ≥1 ε4 allele. Baseline memory, executive function, and language composites represent harmonized cognitive composite scores at baseline (higher values indicate better performance). Vascular risk score reflects a standardised composite vascular risk index (described in Methods). BMI missingness: TARCC missing *n* = 3; EFIGA missing *n* = 3.

Abbreviations: ADNI, Alzheimer's Disease Neuroimaging Initiative; EFIGA, Estudio Familiar de Influencia Genética en Alzheimer; MAP, Rush Memory and Aging Project; NACC, National Alzheimer's Coordinating Center; ROS, Religious Orders Study; TARCC, Texas Alzheimer's Research and Care Consortium; WHICAP, Washington Heights/Inwood Columbia Aging Project; WRAP, Wisconsin Registry for Alzheimer's Prevention.

ADHD‐PRS data distribution is displayed in Figure S1. Associations between ADHD‐PRS and baseline characteristics are shown in Table S3. Individuals with higher ADHD‐PRSs were more likely to be female, have lower educational attainment, and have higher vascular risk scores. There was no significant association between ADHD‐PRS and baseline age or *APOE* ε4 status.

### 3.1 ADHD‐PRS and incident cognitive impairment

Over 10 years of follow‐up, 1347 participants developed incident cognitive impairment, including 1163 transitions from CU to MCI and 184 transitions from CU to AD dementia. The Kaplan–Meier estimated 10‐year cumulative incidence of cognitive impairment was 33.8% (95% CI: 30.7 to 36.8) in the upper ADHD‐PRS tertile and 23.4% (95% CI: 21.4 to 25.4) in the lower tertile (log‐rank *p *< 0.0001). This corresponds to an absolute difference of 10.4 percentage points, or approximately 10 additional cases per 100 individuals over the course of 10 years. Kaplan–Meier curves by ADHD‐PRS strata are shown in Figure 1A. In adjusted Cox models, higher ADHD‐PRS was associated with greater risk of incident cognitive impairment per 1‐SD increase in ADHD‐PRS (hazard ratio [HR] = 1.11, 95% CI: 1.02 to 1.20, *P *= 0.014). Cohort‐specific estimates were not statistically significant and were broadly consistent in direction (Figure 1B), with HR > 1 in all cohorts except one. Results were similar after additional adjustment for education, *APOE* ε4 status, or vascular risk score (Figure 1C). In sensitivity analyses, PRSs for ASD, BD, MDD, and SCZ were not significantly associated with incident cognitive impairment in adjusted models (Figure 2A–H). Although Kaplan–Meier curves suggested separation for MDD‐PRS (Figure 2E), adjusted Cox models did not show a significant association (Figure 2F).

**FIGURE 1:**
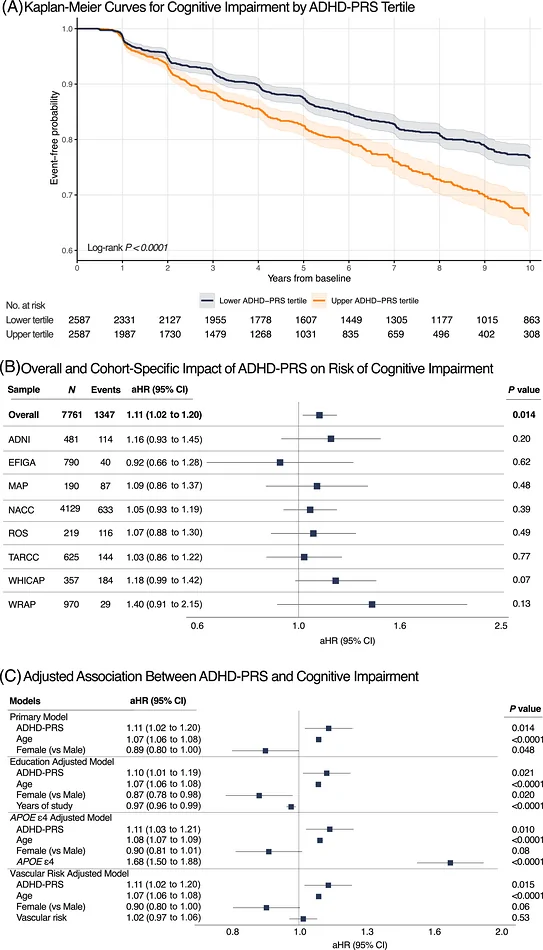
ADHD‐PRS and risk of incident cognitive impairment. Kaplan–Meier curves and Cox proportional hazards models evaluating associations between ADHD‐PRS and incident cognitive impairment (MCI or AD dementia) for up to 10 years of follow‐up. (A) Kaplan–Meier curves comparing lower versus upper ADHD‐PRS tertile. Shaded bands indicate 95% CIs, and the log‐rank *p* values compare tertiles. (B) Overall and cohort‐specific aHRs for ADHD‐PRS. The overall estimate is adjusted for ancestry, baseline age, sex, and cohort, whereas cohort‐specific estimates are adjusted for ancestry, baseline age, and sex. (C) Adjusted associations between ADHD‐PRS and incident cognitive impairment as aHRs per 1‐SD increase in PRS from sequential Cox models with covariates as listed in the figure. Cox models additionally adjusted for ancestry (using the first three PCs) and cohort. ADHD‐PRS, attention‐deficit/hyperactivity disorder polygenic risk score; aHR, adjusted hazard ratio; CI, confidence interval; AD, Alzheimer's disease; MCI, mild cognitive impairment; PC, principal component; ADNI, Alzheimer's Disease Neuroimaging Initiative; EFIGA, Estudio Familiar de Influencia Genética en Alzheimer; MAP, Rush Memory and Aging Project; ROS, Rush Religious Orders Study; NACC, National Alzheimer's Coordinating Center; TARCC, Texas Alzheimer's Research and Care Consortium; WHICAP, Washington Heights/Inwood Columbia Aging Project; WRAP, Wisconsin Registry for Alzheimer's Prevention.

**FIGURE 2:**
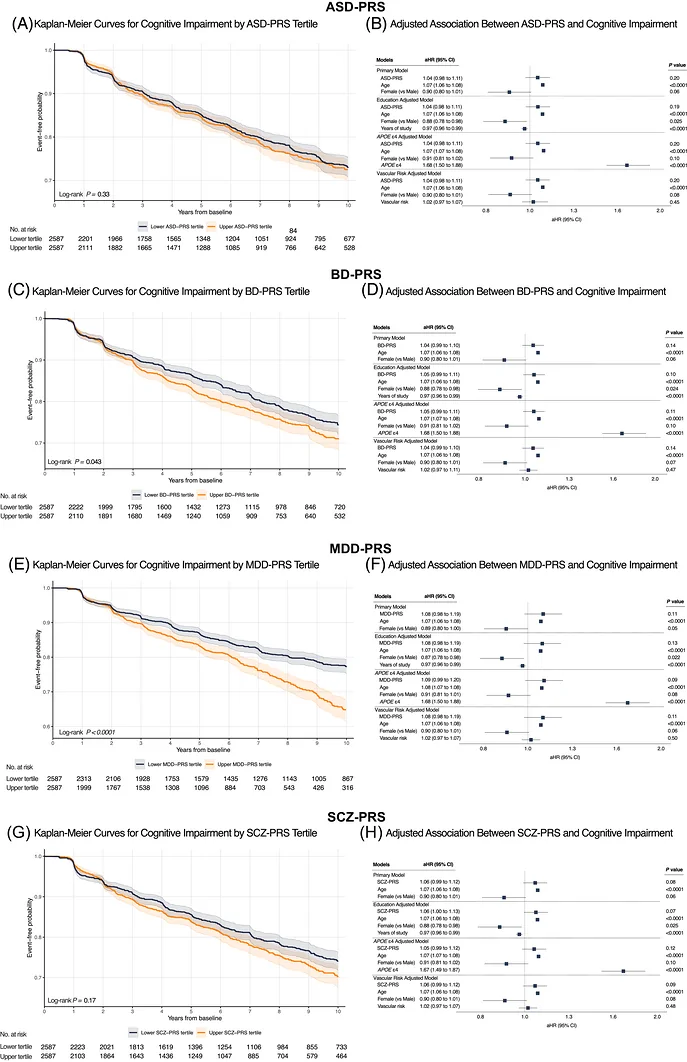
PRS for other psychiatric disorders and risk of incident cognitive impairment. Kaplan–Meier curves and Cox proportional hazards models evaluating associations between psychiatric PRS and incident cognitive impairment (MCI or AD dementia) during up to 10 years of follow‐up. (A, C, E, and G) Kaplan–Meier curves comparing lower versus upper PRS tertile for ASD‐PRS, BD‐PRS, MDD‐PRS, and SCZ‐PRS, respectively. Shaded bands indicate 95% CIs, and the log‐rank *p* values compare tertiles. (B, D, F, and H) show adjusted associations between each PRS and incident cognitive impairment as aHRs per 1‐SD increase in PRS from sequential Cox models with covariates as listed in the figure. Cox models additionally adjusted for ancestry (using the first three PCs) and cohort. PRS, polygenic risk score; ASD, autism spectrum disorder; BD, bipolar disorder; MDD, major depressive disorder; SCZ, schizophrenia; aHR, adjusted hazard ratio; CI, confidence interval; AD, Alzheimer's disease; MCI, mild cognitive impairment; PC, principal component; ADNI, Alzheimer's Disease Neuroimaging Initiative; EFIGA, Estudio Familiar de Influencia Genética en Alzheimer; MAP, Rush Memory and Aging Project; ROS, Rush Religious Orders Study; NACC, National Alzheimer's Coordinating Center; TARCC, Texas Alzheimer's Research and Care Consortium; WHICAP, Washington Heights/Inwood Columbia Aging Project; WRAP, Wisconsin Registry for Alzheimer's Prevention.

Among the 1347 participants with incident MCI or AD dementia, 458 (34.0%) later returned to CU status within the analytic follow‐up window. Of these, 299 (65.3%) were sustained CU reverters who remained CU at all subsequent follow‐up visits, and 159 (34.7%) were transient CU reverters who later reconverted to MCI or AD dementia. After treating sustained CU reverters as non‐events and retaining transient CU reverters as events, 1048 events remained. In this analysis, higher ADHD‐PRS remained associated with incident cognitive impairment (HR = 1.11, 95% CI: 1.01 to 1.22; P = 0.027).

### 3.2 Aβ subsample analyses

A total of 1146 participants had available Aβ status (ADNI *n* = 411, NACC *n* = 436, WRAP *n* = 299). Most Aβ measures were PET‐based (*n* = 766). Overall, 815 (71.1%) participants were Aβ‐negative and 331 (28.9%) were Aβ‐positive. Demographic and clinical variables at Aβ‐baseline can be found in Table S4. ADHD‐PRS was not associated with Aβ positivity after adjustment for age at baseline, sex, study, and ancestry (odds ratio [OR] = 1.04, 95% CI: 0.88 to 1.23, *p *= 0.65). In Cox models anchored at Aβ‐baseline, ADHD‐PRS remained associated with incident cognitive impairment (HR = 1.32, 95% CI: 1.09 to 1.60, *p *= 0.005; Figure 3A,B). There was no evidence of effect modification by Aβ status (ADHD‐PRS × Aβ interaction HR = 0.95, 95% CI: 0.69 to 1.30, *p *= 0.75). Aβ‐stratified estimates are shown in Figure 3C–F.

**FIGURE 3:**
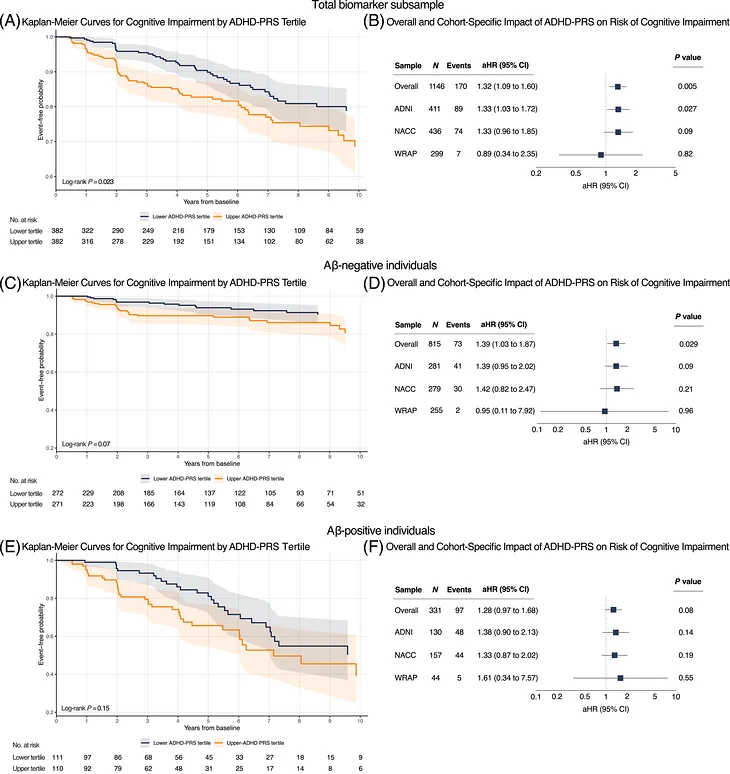
ADHD‐PRS and incident cognitive impairment by Aβ status. Kaplan–Meier curves and Cox proportional hazards models evaluating associations between ADHD‐PRS and incident cognitive impairment (MCI or AD dementia) among participants with available Aβ biomarkers. Baseline was defined as the visit at which Aβ status was ascertained. (A, C, and E) Kaplan–Meier curves comparing lower and upper ADHD‐PRS tertile in total biomarker subsample, Aβ‐negative individuals, and Aβ‐positive individuals, respectively. Shaded bands indicate 95% CIs, and log‐rank *p* value compares tertiles. (B, D, and F) Overall and cohort‐specific Cox model estimates, reported as aHRs per 1‐SD increase in ADHD‐PRS with 95% CIs. Pooled Cox models were adjusted for age, sex, ancestry (using the first three PCs), and cohort. Cohort‐specific Cox models were adjusted for age, sex, and ancestry. ADHD‐PRS, attention‐deficit/hyperactivity disorder polygenic risk score; Aβ, amyloid beta; aHR, adjusted hazard ratio; CI, confidence interval; MCI, mild cognitive impairment; AD, Alzheimer's disease; PC, principal component; ADNI, Alzheimer's Disease Neuroimaging Initiative; NACC, National Alzheimer's Coordinating Center; WRAP, Wisconsin Registry for Alzheimer's Prevention.

### 3.3 ADHD‐PRS and cognition

In linear mixed‐effects models of harmonized cognitive composites, higher ADHD‐PRS was associated with lower baseline performance in memory (*β* = −0.031, 95% CI: −0.046 to −0.016, *p *< 0.0001), executive function (*β* = −0.056, 95% CI: −0.075 to −0.037, *p *< 0.0001), and language (*β* = −0.029, 95% CI: −0.046 to −0.012, *p *= 0.0007) (Table S5). For longitudinal change, ADHD‐PRS was not associated with the rate of change in memory (PRS × time *β* = 0.0017, 95% CI: −0.00039 to 0.00385, *p *= 0.11) or language (PRS × time *β* = 0.00027, 95% CI: −0.00168 to 0.00221, *p *= 0.79) (Figure S2). However, there was a significant interaction between ADHD‐PRS and time for executive function (PRS × time *β* = 0.0040, 95% CI: 0.0021 to 0.0058, *p *< 0.0001), indicating that individuals with higher ADHD‐PRS experienced a less steep decline in executive function over time.

## 4 DISCUSSION

This prospective multicohort analysis of 7761 CU older adults provides evidence that higher ADHD‐PRS, a probabilistic estimate of genetic risk for ADHD, is associated with greater risk of clinically ascertained incident cognitive impairment over 10 years. Baseline Aβ status did not modify this risk in the subsample with available biomarker data. The specificity of this association to ADHD, in contrast to the null findings for four other psychiatric disorders tested within the same analytic framework, suggests that higher ADHD‐PRS confers a distinct and previously underappreciated contribution to late‐life cognitive vulnerability.

Our primary finding that higher ADHD‐PRS associates with progression from CU to MCI/AD aligns with epidemiological studies linking ADHD diagnosis to later‐life cognitive impairment and dementia.^3^, ^4^, ^5^, ^6^, ^7^, ^8^, ^9^, ^10^ Unlike prior observational studies, which relied largely on administrative records, the ADHD‐PRS provides a measure derived from inherited common genetic variation that is not dependent on healthcare utilization or clinical recognition.This is particularly relevant in older cohorts, where ADHD is likely underdiagnosed.^32^ Furthermore, MCI and AD dementia were ascertained through standardized clinical evaluations, providing a more rigorous approach than electronic health record definitions. Participants in the highest ADHD‐PRS tertile had approximately 10 additional cases of cognitive impairment per 100 individuals over 10 years compared with the lowest tertile. This provides a rationale for further work to determine whether individuals with higher ADHD‐PRS or clinically recognized ADHD warrant heightened vigilance for cognitive decline.

Notably, we found no evidence that PRSs for other psychiatric disorders (ASD, BD, MDD, and SCZ) were associated with incident cognitive impairment. This is relevant since psychiatric disorders show substantial genetic overlap,^33^ raising the possibility that a shared transdiagnostic liability could underlie later cognitive decline. Our results do not support that explanation in this sample. Additionally, ADHD is not the only psychiatric condition proposed to increase risk for cognitive decline. The evidence is more extensive for MDD,^34^ and SCZ^35^ and BD^36^ have also been associated with dementia risk, with more limited evidence for ASD.^37^ Importantly, our study evaluated genetic risk captured by PRS rather than clinical diagnoses. For some disorders, particularly MDD, PRS may capture a smaller fraction of risk,^27^ potentially contributing to the null findings. Thus, although only ADHD‐PRS was associated with cognitive impairment in this analytic framework, these results do not preclude clinical associations between other psychiatric disorders and late‐life cognitive decline.

In the biomarker subsample, ADHD‐PRS was not associated with Aβ positivity, and Aβ did not modify the association between ADHD‐PRS and clinical progression. These findings support that higher ADHD‐PRS may be associated with cognitive vulnerability through mechanisms that are at least partially independent of baseline Aβ pathology. Although prior studies reported mixed findings regarding ADHD‐PRS × Aβ interactions,^18^, ^19^ our results do not support a markedly different effect of ADHD‐PRS in individuals above conventional Aβ positivity thresholds. Still, because Aβ accumulation is a continuous process, dichotomizing Aβ for clinical classification may obscure more subtle susceptibility effects.^38^ It remains possible that individuals with higher ADHD‐PRSs experience adverse cognitive consequences at lower levels of Aβ burden, but this would need to be explored in future work.

Higher ADHD‐PRS was associated with lower cognitive performance across memory, executive function, and language domains, without evidence of faster decline over time. Together with prior research showing an association between ADHD‐PRS and lower cognitive performance in structured testing,^15^, ^16^ these findings are consistent with lifelong traits that predate the observation window. Importantly, cognitive performance declined over time across all ADHD‐PRS strata, and individuals with higher ADHD‐PRS consistently performed worse throughout follow‐up. The less steep executive function decline observed among individuals with higher ADHD‐PRS likely reflects a floor effect, whereby lower baseline performance limits measurable further decline rather than indicating a protective trajectory. These findings suggest that persistently lower performance in individuals with higher ADHD‐PRS may place them closer to the threshold at which clinical impairment becomes detectable.

Currently, the mechanisms underlying the association of ADHD and ADHD‐PRS with cognitive impairment in older age remain unclear. Lower educational attainment and higher vascular risk have been proposed as shared factors, but our findings, along with those of prior studies,^6^, ^7^, ^9^, ^18^ suggest that they do not fully explain the association. ADHD may instead confer risk through multiple interacting processes across the lifespan, including long‐standing differences in attention and executive‐control networks^39^ that could reduce cognitive reserve, as well as the cumulative burden of health‐related exposures more common in ADHD, such as sleep disturbance,^40^ substance use,^41^ traumatic brain injury,^42^ obesity,^43^ and poorer physical health.^44^ Disentangling these pathways is challenging, particularly because many relevant exposures fluctuate over decades and may not be well captured in older‐age cohorts. Long‐term prospective studies following childhood‐diagnosed ADHD into later life, with repeated neurocognitive, comorbidity, and biomarker assessments, will be important to clarify whether risk is driven primarily by cumulative exposures, reduced reserve, non‐AD neuropathologies, or their combination.

Findings from this study should be interpreted with consideration of the following limitations. ADHD‐PRS is an indirect measure of common‐variant genetic risk that explains only a modest proportion of variance in clinical ADHD and ADHD‐related traits, estimated at approximately 0.7% to 5.5%,^16^, ^45^ and therefore does not capture the full clinical phenotype in older adults. The ADHD‐PRS captures only one component of ADHD risk and does not directly account for rare genetic variation, environmental exposures, or gene–environment interplay. In addition, the ADHD‐PRS was derived from a GWAS that was predominantly composed of individuals of European ancestry,^15^ and the generalizability across diverse ancestral groups may be limited. Volunteer‐based cohorts may preferentially include individuals with less severe psychiatric illness,^46^ and some cohorts included here excluded or may have underrepresented individuals with significant psychiatric or neurodevelopmental conditions. This may affect the distribution of psychiatric PRS in the analytic sample relative to the general older adult population, particularly reducing representation of individuals at the highest psychiatric common‐variant genetic risk. Biomarker data were limited relative to the full sample. Although we adjusted for vascular risk factors using a harmonized score, we did not systematically adjust for all medical comorbidities that could influence cognitive outcomes, and residual confounding remains possible. Due to data limitations, we were unable to evaluate longitudinal changes in Aβ in relation to ADHD‐PRS and cognitive decline. Finally, since ADHD symptoms can overlap with those of MCI, the possibility that individuals with high ADHD‐PRS may have been misdiagnosed with MCI should be considered.

In conclusion, ADHD‐PRS was associated with incident late‐life cognitive impairment in pooled multicohort analyses of older adults, through mechanisms distinct from AD Aβ pathways. These findings support ADHD genetic risk as a potential contributor to cognitive aging and suggest that the lifespan trajectory of ADHD may intersect with dementia risk through pathways that remain to be clarified. Individuals with ADHD, including older adults in whom the condition has gone unrecognized, may represent an underidentified at‐risk population for whom earlier cognitive monitoring and proactive management of modifiable risk factors could be beneficial. Future studies integrating ADHD phenotyping with biomarker panels and mechanistic analyses will be important for translating these findings into prevention strategies.

## CONFLICT OF INTEREST STATEMENT

Tharick A. Pascoal serves on a Data Safety Monitoring Board for NIH award 5 R01 AG084834 based at the University of Cincinnati. He also has served on a scientific advisory board at Johnson & Johnson. Luis Augusto Rohde has received grant or research support from, served as a consultant to, and served on the speakers’ bureau of Abdi Ibrahim, Abbott, Aché, Adium, Apsen, Bial, Cellera, EMS, Hypera Pharma, Knight Therapeutics, Libbs, Medice, Novartis/Sandoz, Pfizer/Upjohn/Viatris, Shire/Takeda, and Torrent in the last three years. The ADHD and Juvenile Bipolar Disorder Outpatient Programs chaired by Luis Augusto Rohde have received unrestricted educational and research support from the following pharmaceutical companies in the last three years: Novartis/Sandoz and Shire/Takeda. Luis Augusto Rohde has received authorship royalties from Oxford Press and ArtMed. Samuele Cortese has declared reimbursement for travel and accommodation expenses from the Association for Child and Adolescent Central Health (ACAMH) in relation to lectures delivered for ACAMH, the Canadian AADHD Alliance Resource, the British Association of Psychopharmacology, Healthcare Convention and CCM Group team for educational activity on ADHD, and has received honoraria from Medice. Douglas Teixeira Leffa and Brooke S. G. Molina received travel awards to attend the American Professional Society of ADHD and Related Disorders (APSARD) 2024, 2025, and 2026 Annual Meetings, and to attend the 10th World Congress on ADHD. All other authors report no conflicts of interest. Author disclosures are available in the Supporting Information.

## CONSENT

Across all cohorts, written informed consent was provided by participants, and research was conducted in accordance with approved Institutional Review Board protocols. Secondary analysis of these data was approved by the University of Pittsburgh IRB.

## Supporting information




Supporting Information

Supporting Information
