# Supplementary material for: Attention‐deficit/hyperactivity disorder polygenic risk score and incidence of cognitive impairment in older adults: A prospective multicohort study
Source: Alzheimers Dement. 2026 Oct 15;22(10):e71770. doi: 10.1002/alz.71770 (PMC13670426; doi:10.1002/alz.71770)
Supplement: Supplementary file 2 [file ALZ-22-e71770-s001.docx]

**Supplementary Material**

[Supplementary Methods	2](#_Toc234913466)

[Description of included cohorts	2](#_Toc234913467)

[Table S1. Cohort-specific diagnostic criteria	5](#_Toc234913468)

[Table S2. Cohort enrollment and follow-up schedule	7](#_Toc234913469)

[Figure S1. PRS Histograms	8](#_Toc234913470)

[Table S3. Associations of ADHD-PRS with Baseline Demographic and Clinical Characteristics	9](#_Toc234913471)

[Table S4. Demographic and Clinical Characteristics of the Biomarker Subsample at Aβ-Baseline	10](#_Toc234913472)

[Table S5. Associations of ADHD-PRS with Baseline and Longitudinal Change in Cognition in Linear Mixed-Effects Models	12](#_Toc234913473)

[Figure S2. Associations Between ADHD-PRS and Cognitive Trajectories	14](#_Toc234913474)

[References	15](#_Toc234913475)

# Supplementary Methods

## Description of included cohorts

**Alzheimer’s Disease Neuroimaging Initiative (ADNI)**

ADNI is a longitudinal, multicenter observational program established at over 60 clinical sites in the USA and Canada to develop and validate clinical, imaging, genetic, and biochemical biomarkers for the early detection and tracking of Alzheimer’s disease (AD) and for use as outcomes in AD clinical trials. ADNI follows participants across the cognitive spectrum using harmonized, repeated assessments over time. Data collection includes serial clinical and neuropsychological evaluations, structural MRI, PET imaging, genetic data, and biospecimen collection (including optional lumbar puncture), enabling integrated analyses of clinical trajectories and biomarker change. Eligible participants are 55 to 90 years of age (inclusive), willing to undergo all study procedures, free from any significant neurologic disease and in good health. Participants are classified as mild cognitive impairment (MCI) or AD dementia based on comprehensive clinical, cognitive, and functional assessments. Further information can be found at [http://adni.loni.usc.edu](http://adni.loni.usc.edu/) and in prior publications.^1^

**Estudio Familiar de Influencia Genetica en Alzheimer (EFIGA)**

EFIGA is a family-based study that began recruitment in 1998 to investigate genetic contributors to late-onset AD (LOAD) in families of Caribbean Hispanic ancestry. The cohort includes multiplex families (typically families with ≥2 relatives affected by AD) and their relatives who are considered at risk. Recruitment was conducted through the Taub Institute/Columbia University in New York City (including community outreach in Washington Heights-Inwood) and through collaborating clinics in the Dominican Republic. Participants underwent standardized neurological and neuropsychological evaluations. Dementia severity is rated using the Clinical Dementia Rating (CDR), and case status are defined using NINCDS-ADRDA criteria for probable or possible LOAD. When available, brain imaging and laboratory information are reviewed to support diagnostic classification. Further information can be found at <http://www.cumc.columbia.edu/adrc/investigators> and in prior publications.^2,3^

**National Alzheimer’s Coordinating Center (NACC)/NIA Alzheimer’s Disease Research Centers (ADRCs)**

NACC aggregates and standardizes participant-level clinical and neuropathology information collected at NIA-funded ADRCs across the United States. This is done primarily through the Uniform Data Set (UDS) annual evaluation framework, including demographics and medical history, medications, neurological/functional status, neuropsychological testing, and clinician-determined, criteria-based diagnoses. The UDS captures longitudinal phenotyping spanning cognitively normal individuals, MCI, and dementia, reflecting each ADRC’s Clinical Core enrollment. ADRCs implement standard diagnostic criteria to classify individuals with major and mild neurocognitive disorders (DSM-5-TR) and incorporates NIAA-AA Criteria which emphasize a biological continuum from preclinical stages to dementia. The ADRC cohort contributing to ADGC/ADSP includes (i) autopsy-confirmed and clinically confirmed AD cases, (ii) cognitively normal elders (CNEs) with complete neuropathology data who were ≥60 years at death, and (iii) living CNEs evaluated using the UDS protocol who were documented not to have MCI and were typically 60-100 years old at assessment. Further information can be found at <https://www.naccdata.org/> and in prior publications.^4,5^

**Religious Orders Study/Memory and Aging Project (ROS/MAP)**

The Religious Orders Study (ROS) and the Rush Memory and Aging Project (MAP) are two ongoing, longitudinal clinical-pathologic cohorts run by the Rush Alzheimer’s Disease Center that share a large common “core” of measures and were created to study common chronic conditions of aging and the neuropathologic substrates of late-life cognitive impairment and dementia. ROS began clinical evaluations in 1994 and enrolls older members of Catholic religious communities (nuns, priests, and brothers) from across the United States; MAP began in 1997 and enrolls older community-dwelling adults largely recruited from retirement communities and senior housing in the Chicago metropolitan region/northeastern Illinois. In both studies, participants without known dementia are enrolled and agree to structured annual clinical and cognitive evaluations (with blood draws in many participants) and to post-mortem organ donation (brain donation in ROS; brain plus additional tissues such as spinal cord and selected nerves/muscles in MAP). Clinical diagnoses are generated using a standardized diagnostic process shared across ROS and MAP, including algorithmic decision support designed to emulate expert judgment and clinician adjudication. NINCDS/ADRDA criteria are used for diagnosis of AD dementia, and the diagnosis of MCI is given to individuals who are judged to have cognitive impairment by a neuropsychologist but do not meet accepted criteria for dementia by a clinician. Participants are ultimately classified (e.g., cognitively unimpaired, MCI, dementia/AD) based on annual evaluations and final review of ante-mortem information. Further information can be found at <https://www.rushu.rush.edu/research/departmental-research/rush-alzheimers-disease-center/radc-research/epidemologic-research>

and in prior publications.^6-8^

**Washington Heights/Inwood Columbia Aging Project (WHICAP)**

WHICAP is a community-based, prospective cohort launched in 1992 to study cognitive aging and dementia among older adults living in northern Manhattan (Washington Heights and Inwood), with a particular emphasis on identifying environmental, medical, and biologic predictors of AD risk and progression in a multi-ethnic population. Participants were originally recruited as a probability sample of Medicare beneficiaries aged ≥65 years residing in the Washington Heights-Inwood area (described in early WHICAP publications as a geographically defined northern Manhattan catchment), and did not have known dementia at enrollment. Across recruitment waves, WHICAP has enrolled thousands of participants and includes substantial representation of non-Hispanic White, African American, and Caribbean Hispanic (Dominican) individuals. At each evaluation, participants complete a structured neuropsychological test battery and medical interview; clinical diagnoses are determined by expert consensus and commonly referenced to NINCDS-ADRDA criteria for possible/probable/definite AD. MCI classification is assigned based on AAN (2001) criteria. WHICAP also supports longitudinal investigation of a wide range of aging-related factors (e.g., emotional health, functional independence, vascular measures, and selected blood-based biomarkers) and includes neuroimaging sub-studies (e.g., MRI and PET) in subsets of participants. Further information can be found at <https://www.maelstrom-research.org/mica/individual-study/whicap> and in prior publications.^9,10^

**Wisconsin Registry for Alzheimer’s Prevention (WRAP)**

WRAP is an ongoing longitudinal observational cohort that began enrollment in 2001 and is enriched for individuals at increased AD risk due to a parental history of probable AD dementia. WRAP enrolls adults 40-65 years at baseline who are free of dementia at study entry. Participants return for a second visit approximately 4 years after baseline, with subsequent follow-up visits occurring about every 2 years. At each visit, WRAP collects a comprehensive cognitive test battery, self-reported medical and lifestyle information (including domains such as physical/cognitive activity, sleep, and mood), and blood for laboratory measures and multi-omics; subsets additionally undergo neuroimaging and CSF collection for biomarker assessments. Clinical diagnoses are performed based on National Institute on Aging-Alzheimer's Association criteria. Further information can be found at <https://wrap.wisc.edu/> and in prior publications.^11^

**Texas Alzheimer’s Research and Care Consortium (TARCC)**

TARCC is a multi-center Texas collaboration that enrolls older adults (≥50), comprised of cognitively unimpaired subjects, MCI patients, and AD patients, and follows them longitudinally with standardized clinical, neurologic, and neuropsychological assessments. Participants are examined at baseline and typically return annually. Clinical diagnoses are assigned via site consensus (at minimum including a physician and neuropsychology expertise) using established diagnostic criteria including NINCDS/ADRDA criteria. MCI is assigned based on clinical indicators, specifically the presence of cognitive impairment but not dementia. Further information can be found at <http://www.txalzresearch.org/> and in prior publications.^12^

# Table S1. Cohort-specific diagnostic criteria

| **Cohort** | **CU** | **MCI** | **AD Dementia** |
| --- | --- | --- | --- |
| **ADNI** | No significant cognitive or functional impairment; criteria generally included MMSE 24-30, CDR global = 0, CDR memory box = 0, and normal Logical Memory II delayed recall, with slight variation across ADNI phases. | Subjective memory concern, abnormal Logical Memory II delayed recall, MMSE 24-30, CDR global = 0.5 with memory box >=0.5, preserved function, and no dementia, with slight variation across ADNI phases. | AD dementia meeting NINCDS-ADRDA criteria with mild-stage MMSE/CDR thresholds, with slight variation across ADNI phases. |
| **EFIGA** | No cognitive impairment after clinical evaluation; no dementia or MCI. | MCI classification assigned based on AAN 2001 criteria. | AD dementia based on established research criteria, including NINCDS-ADRDA criteria. |
| **ROS/MAP** | No cognitive impairment after clinical evaluation; no dementia or MCI. | Neuropsychologist judged cognitive impairment, but clinician determined dementia criteria were not met. | AD dementia based on NINCDS/ADRDA criteria, including decline with memory plus another cognitive domain affected. |
| **NACC / ADRCs** | Generally CDR = 0 and/or normal neuropsychological testing. | Concern about cognitive change, impairment in at least one domain, largely preserved independence, and no dementia. | AD dementia diagnosed using 2011 NIA-AA criteria, with probable or possible AD etiology assigned through the clinician diagnosis process. |
| **TARCC** | Normal cognition/normal control by consensus clinical review; generally no cognitive complaints, normal CDR/CDR-SB, and testing within normal limits. | Consensus diagnosis based on clinical indicators, specifically the presence of cognitive impairment but no dementia. | Consensus AD diagnosis using NINCDS-ADRDA AD criteria. |
| **WHICAP** | No cognitive impairment after clinical evaluation; no dementia or MCI. | MCI classification assigned based on AAN 2001 criteria. | Dementia by consensus conference using DSM criteria; probable/possible AD by NINCDS-ADRDA criteria. |
| **WRAP** | No cognitive impairment after clinical evaluation; no dementia or MCI. | NIA-AA MCI criteria requiring concern about cognitive change, impairment in one or more domains, and no dementia. | Consensus AD diagnosis using NINCDS-ADRDA AD criteria. |

Diagnostic criteria used to classify CU status, MCI, and AD dementia across contributing ADSP cohorts.

Abbreviations: AD, Alzheimer’s disease; ADNI, Alzheimer’s Disease Neuroimaging Initiative; ADRC, Alzheimer’s Disease Research Center; AAN, American Academy of Neurology; CDR, Clinical Dementia Rating; CU, cognitively unimpaired; EFIGA, Estudio Familiar de Influencia Genetica en Alzheimer; MCI, mild cognitive impairment; MMSE, Mini-Mental State Examination; NACC, National Alzheimer’s Coordinating Center; NIA-AA, National Institute on Aging-Alzheimer’s Association; NINCDS-ADRDA, National Institute of Neurological and Communicative Disorders and Stroke-Alzheimer’s Disease and Related Disorders Association; ROS/MAP, Religious Orders Study/Memory and Aging Project; TARCC, Texas Alzheimer’s Research and Care Consortium; WHICAP, Washington Heights-Inwood Columbia Aging Project; WRAP, Wisconsin Registry for Alzheimer’s Prevention.

# Table S2. Cohort enrollment and follow-up schedule

| **Cohort** | **Start of recruitment** | **Follow-up** |
| --- | --- | --- |
| **ADNI** | ADNI 1 started in 2004; ADNI GO in 2009; ADNI 2 in 2011; ADNI 3 in 2016; ADNI 4 in 2022. | Protocol-specific repeated follow-up; generally semiannual to annual, depending on ADNI phase and diagnostic group. |
| **EFIGA** | EFIGA was initiated in 1998. | Participants were followed every 2 years. |
| **ROS/MAP** | ROS enrollment began in 1994; MAP enrollment began in 1997. | Participants underwent annual clinical evaluations. |
| **NACC / ADRCs** | NACC itself was established before UDS; UDS standardized data collection began in 2005. | Participants contributed approximately annual UDS visits. |
| **TARCC** | The standardized research cohort began in 2005. | Participants contributed approximately annual visits. |
| **WHICAP** | WHICAP began in 1992, with later recruitment waves in 1999 and 2009. | Participants were evaluated approximately every 2 years. |
| **WRAP** | WRAP enrollment began in 2001. | Participants returned approximately 4 years after baseline for visit 2 and every 2 years thereafter. |

Enrollment and typical follow-up schedule for each contributing cohort.

Abbreviations: ADNI, Alzheimer’s Disease Neuroimaging Initiative; ADRC, Alzheimer’s Disease Research Center; EFIGA, Estudio Familiar de Influencia Genetica en Alzheimer; NACC, National Alzheimer’s Coordinating Center; ROS/MAP, Religious Orders Study/Memory and Aging Project; TARCC, Texas Alzheimer’s Research and Care Consortium; UDS, Uniform Data Set; WHICAP, Washington Heights-Inwood Columbia Aging Project; WRAP, Wisconsin Registry for Alzheimer’s Prevention.

# Figure S1. PRS Histograms

Distribution of PRS values (z-scored) in the sample.

Abbreviations: PRS, polygenic risk score; ADHD, attention-deficit/hyperactivity disorder; ASD, autism spectrum disorder; BD, bipolar disorder; MDD, major depressive disorder; SCZ, schizophrenia.

# Table S3. Associations of ADHD-PRS with Baseline Demographic and Clinical Characteristics

| **Continuous variables** | **β** | **SE** | **95% CI** | **t** | ***P*** |
| --- | --- | --- | --- | --- | --- |
| **Age, y** | -0.011 | 0.120 | [-0.245, 0.222] | -0.096 | 0.92 |
| **Years of education** | -0.304 | 0.061 | [-0.422, -0.185] | -5.009 | <0.0001 |
| **Vascular risk score** | 0.077 | 0.019 | [0.040, 0.115] | 4.049 | <0.0001 |
| **BMI** | 0.478 | 0.092 | [0.298, 0.659] | 5.194 | <0.0001 |
| **Categorical variables** | **OR** | **SE** | **95% CI** | **T** | ***P*** |
| **Sex** | 1.137 | 0.037 | [1.058, 1.223] | 3.482 | 0.0005 |
| ***APOE* ε4 carrier status** | 1.057 | 0.038 | [0.981, 1.139] | 1.457 | 0.15 |
| **Hypertension** | 1.032 | 0.051 | [0.933, 1.141] | 0.605 | 0.54 |
| **Diabetes** | 1.212 | 0.045 | [1.110, 1.324] | 4.275 | <0.0001 |
| **Heart disease** | 1.051 | 0.040 | [0.971, 1.138] | 1.237 | 0.21 |
| **Stroke** | 0.978 | 0.068 | [0.857, 1.117] | -0.329 | 0.74 |

Associations between ADHD-PRS (per 1-SD increase) and baseline characteristics. Continuous outcomes were modeled with linear regression and are reported as β with SE, 95% CI, t, and two-sided *P*. Binary outcomes were modeled with logistic regression and are reported as OR with corresponding SE, 95% CI, t, and two-sided *P* value. For binary outcomes, ORs reflect the odds of being female (vs male), being an *APOE* ε4 carrier (≥1 ε4 allele; vs non-carrier), and having the condition present (vs absent) for hypertension, diabetes, heart disease, and stroke. All models included ancestry adjustment using the first three genetic principal components. Models where the outcome was age or sex were adjusted for ancestry only, while all other models were additionally adjusted for baseline age and sex.

Abbreviations: ADHD-PRS, attention-deficit/hyperactivity disorder polygenic risk score; SE, standard error; CI, confidence interval; OR, odds ratio; BMI, body mass index.

# Table S4. Demographic and Clinical Characteristics of the Biomarker Subsample at Aβ-Baseline

|  | **Total sample (N=1,146)** | **ADNI (N=411)** | **NACC (N=436)** | **WRAP (N=299)** |
| --- | --- | --- | --- | --- |
| **Age at baseline, mean (SD), y** | 72.07 (8.37) | 74.76 (6.34) | 75.34 (6.50) | 63.61 (7.39) |
| **Sex, No. (%)** |  |  |  |  |
| Female | 676 (59.0%) | 219 (53.3%) | 257 (58.9%) | 200 (66.9%) |
| Male | 470 (41.0%) | 192 (46.7%) | 179 (41.1%) | 99 (33.1%) |
| **Race, No. (%)** |  |  |  |  |
| American Indian or Alaska Native | 7 (0.6%) | 1 (0.2%) | 6 (1.4%) | 0 (0.0%) |
| Asian | 9 (0.8%) | 6 (1.5%) | 2 (0.5%) | 1 (0.3%) |
| Black or African American | 104 (9.1%) | 27 (6.6%) | 69 (15.8%) | 8 (2.7%) |
| Native Hawaiian or Other Pacific Islander | 0 (0.0%) | 0 (0.0%) | 0 (0.0%) | 0 (0.0%) |
| White | 1,016 (88.7%) | 371 (90.3%) | 356 (81.7%) | 289 (96.7%) |
| Other/Unknown/More than one race | 10 (0.9%) | 6 (1.5%) | 3 (0.7%) | 1 (0.3%) |
| **Ethnicity, No. (%)** |  |  |  |  |
| Hispanic or Latino | 54 (4.7%) | 16 (3.9%) | 36 (8.3%) | 2 (0.7%) |
| Not Hispanic or Latino | 1,088 (94.9%) | 392 (95.4%) | 399 (91.5%) | 297 (99.3%) |
| Not available | 4 (0.3%) | 3 (0.7%) | 1 (0.2%) | 0 (0.0%) |
| **Education, mean (SD), y** | 16.23 (2.92) | 16.44 (2.65) | 15.76 (3.14) | 16.61 (2.89) |
| ***APOE* ε4, No. carriers (%)** | 394 (34.4%) | 114 (27.7%) | 159 (36.5%) | 121 (40.5%) |
| **Vascular risk factors** |  |  |  |  |
| Vascular risk score, mean (SD) | -0.40 (1.10) | -0.93 (0.71) | 0.14 (1.08) | -0.46 (1.19) |
| Hypertension, No. (%) | 716 (62.5%) | 205 (49.9%) | 399 (91.5%) | 112 (37.5%) |
| Diabetes, No. (%) | 132 (11.5%) | 15 (3.6%) | 79 (18.1%) | 38 (12.7%) |
| Heart disease, No. (%) | 235 (20.5%) | 2 (0.5%) | 147 (33.7%) | 86 (28.8%) |
| Stroke, No. (%) | 50 (4.4%) | 7 (1.7%) | 24 (5.5%) | 19 (6.4%) |
| BMI (SD) | 27.55 (5.35) | 27.19 (4.86) | 27.14 (5.22) | 28.63 (6.00) |
| **Composite score at baseline, mean (SD)** |  |  |  |  |
| Memory composite | 0.86 (0.49) | 0.84 (0.46) | 0.70 (0.49) | 1.12 (0.43) |
| Executive function composite | 0.83 (0.58) | 0.74 (0.48) | 0.70 (0.60) | 1.13 (0.55) |
| Language composite | 0.78 (0.54) | 0.78 (0.53) | 0.82 (0.54) | 0.73 (0.56) |
| **Aβ modality** |  |  |  |  |
| PET | 766 (66.8%) | 339 (82.5%) | 128 (29.4%) | 299 (100%) |
| Fluid | 380 (33.2%) | 72 (17.5%) | 308 (70.6%) | 0 (0%) |
| **Aβ status** |  |  |  |  |
| Negative | 815 (71.1%) | 281 (68.4%) | 279 (64%) | 255 (85.3%) |
| Positive | 331 (28.9%) | 130 (31.6%) | 157 (36%) | 44 (14.7%) |

Values are mean (SD) for continuous variables and No. (%) for categorical variables. Percentages are calculated within each cohort. Age and education are in years (y); BMI is in kg/m². *APOE* ε4 carriers are participants with ≥1 ε4 allele. Baseline memory, executive function, and language composites represent harmonized cognitive composite scores at baseline (higher values indicate better performance). Vascular risk score reflects a standardized composite vascular risk index (described in Methods).

Abbreviations: ADNI, Alzheimer’s Disease Neuroimaging Initiative; NACC, National Alzheimer’s Coordinating Center; WRAP, Wisconsin Registry for Alzheimer’s Prevention.

# Table S5. Associations of ADHD-PRS with Baseline and Longitudinal Change in Cognition in Linear Mixed-Effects Models

| **Cognitive Domain** | **β** | **SE** | **95% CI** | **t** | **N** | **DF** | **P value** |
| --- | --- | --- | --- | --- | --- | --- | --- |
| **ADHD-PRS × time** | | | | | | | |
| Memory | 0.002 | 0.001 | [-3.94x10^-4^, 0.004] | 1.596 | 7757 | 5261.290 | 0.11 |
| Executive function | 0.004 | 9.60x10^-4^ | [0.002, 0.006] | 4.130 | 7678 | 4393.072 | <0.0001 |
| Language | 2.67x10^-4^ | 9.91x10^-4^ | [-0.002, 0.002] | 0.270 | 7752 | 5062.095 | 0.79 |
| **ADHD-PRS** | | | | | | | |
| Memory | -0.031 | 0.008 | [-0.046, -0.016] | -4.054 | 7757 | 7759.301 | <0.0001 |
| Executive function | -0.056 | 0.010 | [-0.075, -0.037] | -5.780 | 7678 | 7642.300 | <0.0001 |
| Language | -0.029 | 0.009 | [-0.046, -0.012] | -3.376 | 7752 | 7813.159 | 0.0007 |

Linear mixed-effects model results for harmonized cognitive composite scores in memory, executive function, and language. The table reports the main effect of ADHD-PRS (association with baseline cognitive performance) and the ADHD-PRS×time interaction (association with rate of change over follow-up). Mixed-effects models included fixed effects for ADHD-PRS, time from baseline, and the ADHD-PRS×time interaction, with adjustment for baseline age, sex, ancestry (using the first three PCs), and cohort. Models included participant-specific random intercepts and random slopes for time. Analyses were restricted using the prespecified precision filter to retain harmonized composite values with SE≤0.6. Estimates are reported as β per 1-SD increase in PRS with corresponding SE, 95% CI, t statistic, and two-sided *P* value. N denotes the number of participants contributing to each model.

Abbreviations: ADHD, attention-deficit/hyperactivity disorder; PRS, polygenic risk score; SE, standard error; CI, confidence interval; PC, principal component; DF, degrees of freedom.

# Figure S2. Associations Between ADHD-PRS and Cognitive Trajectories

Predicted domain-specific cognitive trajectories derived from linear mixed-effects models of harmonized cognitive composite scores (memory, executive function, language) over up to 10 years of follow-up. Plots display model-predicted composite scores across years from baseline for ADHD-PRS strata, and shaded bands indicate 95% CIs. Panels A, B and C show predicted trajectories by ADHD-PRS strata for memory, executive function, and language, respectively. Mixed-effects models included fixed effects for ADHD-PRS, time from baseline, and the ADHD-PRS×time interaction, with adjustment for baseline age, sex, ancestry (using the first three PCs), and cohort. Models included participant-specific random intercepts and random slopes for time.

Abbreviations: ADHD-PRS, attention-deficit/hyperactivity disorder polygenic risk score; CI, confidence interval; PC, principal component.

# References

1.	Petersen, R.C.*, et al.* Alzheimer's Disease Neuroimaging Initiative (ADNI): clinical characterization. *Neurology* **74**, 201-209 (2010).

2.	Vardarajan, B.N.*, et al.* Age-Specific Incidence Rates for Dementia and Alzheimer Disease in NIA-LOAD/NCRAD and EFIGA Families: National Institute on Aging Genetics Initiative for Late-Onset Alzheimer Disease/National Cell Repository for Alzheimer Disease (NIA-LOAD/NCRAD) and Estudio Familiar de Influencia Genetica en Alzheimer (EFIGA). *JAMA Neurology* **71**, 315-323 (2014).

3.	Eissman, J.M.*, et al.* Biological age acceleration associates with Alzheimer's disease plasma biomarker levels. *Alzheimers Dement* **22**, e71005 (2026).

4.	Beekly, D.L.*, et al.* The National Alzheimer's Coordinating Center (NACC) database: the Uniform Data Set. *Alzheimer Dis Assoc Disord* **21**, 249-258 (2007).

5.	Chan, K.C.G., Xia, F. & Kukull, W.A. NACC data: Who is represented over time and across centers, and implications for generalizability. *Alzheimers Dement* **21**, e70657 (2025).

6.	Bennett, D.A.*, et al.* The Rush Memory and Aging Project: study design and baseline characteristics of the study cohort. *Neuroepidemiology* **25**, 163-175 (2005).

7.	Bennett, D.A.*, et al.* Overview and findings from the rush Memory and Aging Project. *Curr Alzheimer Res* **9**, 646-663 (2012).

8.	Bennett, D.A.*, et al.* Selected findings from the Religious Orders Study and Rush Memory and Aging Project. *J Alzheimers Dis* **33 Suppl 1**, S397-403 (2013).

9.	Tang, M.X.*, et al.* Incidence of AD in African-Americans, Caribbean Hispanics, and Caucasians in northern Manhattan. *Neurology* **56**, 49-56 (2001).

10.	Manly, J.J.*, et al.* Implementing diagnostic criteria and estimating frequency of mild cognitive impairment in an urban community. *Arch Neurol* **62**, 1739-1746 (2005).

11.	Johnson, S.C.*, et al.* The Wisconsin Registry for Alzheimer's Prevention: A review of findings and current directions. *Alzheimers Dement (Amst)* **10**, 130-142 (2018).

12.	Waring, S.C.*, et al.* The Texas Alzheimer's Research Consortium longitudinal research cohort: study design and baseline characteristics. *Texas Public Health Journal* **60**(2008).
